# Executive functions in daily living skills: A study in adults with autism spectrum disorder

**DOI:** 10.3389/fpsyg.2023.1109561

**Published:** 2023-04-11

**Authors:** Jo A. Yon-Hernández, Ricardo Canal-Bedia, Dominika Z. Wojcik, Laura García-García, Clara Fernández-Álvarez, Stefanny Palacio-Duque, Manuel A. Franco-Martín

**Affiliations:** ^1^Instituto Universitario de Integración en la Comunidad, InFoAutismo, Universidad de Salamanca, Salamanca, Spain; ^2^Instituto de Investigación Biomédica de Salamanca (IBSAL), Salamanca, Spain; ^3^Zamora Hospital (Complejo Asistencial de Zamora), Zamora, Spain

**Keywords:** autism spectrum disorder, schizophrenia spectrum disorder, executive functions, ASD core symptoms, daily living skills, intelligence quotient

## Abstract

**Introduction:**

Adults with autism and adults with schizophrenia show difficulties in adaptive skills, especially those related to daily functioning. Some studies suggest that adaptive skills are associated with deficits in executive functions (EF), while others indicate that intelligence quotient (IQ) might also play a role. Literature suggests that autistic symptoms further affect adaptive skills. The interest of the current study, therefore, was to explore to what extent IQ, EFs as well as core autistic symptoms predict adaptive skills.

**Methods:**

To do this, 25 controls, 24 adults with autism, and 12 with schizophrenia were assessed on IQ (Wechsler Adult Intelligence Scale), and executive functioning. The EF was measured with neuropsychological tasks (inhibition, updating, and task switching) and with the Dysexecutive-Spanish Questionnaire (DEX-Sp) which assessed everyday life EF problems. Core ASD symptoms were measured using the Autism Diagnostic Observation Schedule, the Autism Spectrum Quotient-Short version (AQ-S), and the Repetitive Behavior Questionnaire – 3 (RBQ-3).

**Results:**

The results indicated EF difficulties in both, autism and schizophrenia. The IQ explained a high percentage of the variance found in adaptive skills, but only in the autism group. We can conclude, therefore, that high IQ is associated with low adaptive skills levels and EFs affect adaptive functioning in people with autism; however, this does not explain the difficulties in adaptive functioning in the schizophrenia group. Core features of autism assessed with self-report questionnaires (but not the ADOS-2) predicted low scores on the adaptive skills, only in the autism group.

**Discussion:**

Both EF measures predicted adaptive skills scores in autism, but not in schizophrenia. Our results suggest that different factors affect the adaptive functioning in each disorder. For instance, the EFs should be a central focus for improvement, especially for individuals with autism.

## Introduction

Autism spectrum disorder (ASD) is a condition characterized by the early onset of difficulties in social communication, restrictive, repetitive behaviors or interests, and atypical sensory sensitivity (American Psychiatric Association, 2013). Autism is a lifelong disorder with a great variability in the presentation and severity of symptoms that change throughout the life span. Among others, symptom severity, level of intellectual functioning, language competence (Lord et al., 2020), executive functioning (EF) (Iversen and Lewis, 2021) as well as shared or co-occurring symptomatology with disorders such as schizophrenia (Lugo-Marín et al., 2019), have been highlighted as important factors that affect the expression of variability in ASD. Indeed, the long-term prognosis of functional and adaptive outcomes for people with ASD is highly dependent on the degree of impairment of these factors, along with the support they receive throughout their life (Hyman et al., 2020). Thus, the main aim of the current study is to examine to what extent variables such as intellectual ability, EFs, and core autism symptoms predict adaptive functioning outcomes for autistic people. Furthermore, we included a clinical group with schizophrenia to examine if the aforementioned variables predict adaptive behavior in ASD specifically or are common in both, ASD and schizophrenia. Ultimately, the identification of key factors that influence adaptive functioning could help professionals design and establish adult-specific therapeutic goals for each condition, as well as the support they may need to achieve desired outcomes. Thus, the motivation behind comparing adults with ASD and schizophrenia is to provide evidence-based and individualized services to improve daily living skills (DLS) according to the different profiles that each group may present on these specific factors, IQ and EFs.

Moreover, it has been demonstrated that adaptive functioning, which refers to the ability to successfully meet the demands of daily life (Sparrow et al., 2005), is particularly impaired in ASD compared to what would be expected for their age, language development, or level of intellectual functioning. It has also been noted that the greatest difficulties in ASD are in socialization and communication skills, followed by difficulties in DLS, such as meal preparation, self-care routines, doing laundry, etc. (Saulnier and Klaiman, 2022). In general, it is assumed that this profile is characteristic of autism, as limitations in the areas of socialization and communication are consistent with the diagnostic criteria of persistent impairments in communication and social interaction (Kenworthy et al., 2010).

A recent study has found that the lesser the impairment in social communication in ASD, the better social and adaptive functioning in adulthood (Tillmann et al., 2019). However, empirical studies have shown that the development of adaptive skills in ASD does not occur at the same pace as that of the typically developing individuals (Pugliese et al., 2016). Adaptive deficits in individuals with ASD increase rather than decrease with age (Klin et al., 2007; Kanne et al., 2011; Pugliese et al., 2015; Chatham et al., 2018; Tillmann et al., 2019). In addition, some factors can affect the outcomes of long-term adaptive functioning, for example, precocity in language development. Children with ASD who start speaking their first words before the age of two, and who make flexible sentences by the age of three, have better social and adaptive functioning as adults (Anderson et al., 2009).

Intellectual functioning, as measured by the intelligence quotient (IQ), is also a factor that should be considered in the study of adaptive skills in ASD. Evidence indicates that an IQ above 70 in adulthood is often associated with better adaptive outcomes. Individuals with greater cognitive deficits or intellectual disability, on the other hand, show greater difficulties in daily life, both short-and long-term (Kanne et al., 2011). This suggests that intellectual deficits indicate worse prognosis in adaptive functioning (Howlin et al., 2013; Jónsdóttir et al., 2018). However, even if there is a significant negative correlation between IQ and adaptive functioning in any age group, adaptive functioning scores are always lower than IQ scores, and the gap between IQ and adaptive functioning is larger in individuals with ASD with average or high IQ than in individuals with lower IQ or with intellectual disabilities (Pugliese et al., 2015; Kraper et al., 2017; Chatham et al., 2018; Tillmann et al., 2019).

The explanation of this significant gap between average IQ and low adaptive behavior has led researchers to think that factors such as the level of social support (Farley et al., 2009), the severity of autism symptoms (Golya and McIntyre, 2018) or the presence of psychiatric co-occurring conditions (Kraper et al., 2017) may all play an important role. To date, little research has been conducted to analyze this gap and there is even more scarce research in adulthood. It is therefore imperative to understand the impact that other variables have on adaptive functioning in adult life because of their role in optimizing personal independence and in maintaining satisfactory social relationships, as well as achieving employment (Howlin et al., 2013; Morrison et al., 2020; Baker et al., 2021).

One of the prominent approaches, which has received growing attention over the past decades, considers EFs as intervening variables in the achievement of optimal adaptive functioning. EF is a broad term that encompasses higher-order cognitive processes and behavioral competencies that serve a general purpose of self-regulation. It involves working memory, planning, cognitive flexibility, inhibition, etc. (Hill, 2004), all of which enable a person to perform intentional actions or to initiate appropriate behaviors or responses (Lezak et al., 2004). Historically, EF has been used to explain the core symptoms of autism not directly related to deficits in social communication, such as inflexibility or insistence on routines (Damasio and Maurer, 1978; Pennington and Ozonoff, 1996). However, difficulties in EF have been correlated to difficulties in emotional and social regulation, as well as in peers and adult-child interactions (Blair and Razza, 2007). Nonetheless, the outcomes of studies that have attempted to assess the relationship between EF and social regulation in ASD are mixed, with this relationship being strong for some aspects of EF but not for others. Thus, there are still many questions about whether executive dysfunction could be a diagnostic marker of ASD (Panerai et al., 2014). A recent meta-analysis confirmed that there is a broad executive dysfunction in ASD, which is relatively stable across development (Demetriou et al., 2018). However, evidence for specific EF subdomains in adults with ASD is much weaker and usually research into EFs in this group focuses on one or two specific subdomains.

Stemming from the idea that EF is linked to adaptive behavior, a large body of studies has identified that EFs serve as predictors of limitations in adaptive behavior in children, adolescents, and adults with ASD with average or high IQ (Gilotty et al., 2002; Peterson et al., 2015; Pugliese et al., 2015; Davids et al., 2016; Pugliese et al., 2016; Wallace et al., 2016). It has also been found that children with ASD who have intellectual disability show difficulties in EF (Panerai et al., 2014; Tsermentseli et al., 2018). Only a few studies have reported age-related improvements in EF skills (Happé et al., 2006; Pellicano, 2010), while others report no improvement (Ozonoff and McEvoy, 1994). Thus, while EF competence in ASD seems to improve throughout childhood and adolescence, similarly to what happens with adaptive functioning, executive abilities develop at a much slower pace, and it is unclear if they remain impaired in adulthood.

Another important variable to consider when examining any clinical condition is the co-occurrence or shared symptomatology with other clinical groups. Interestingly, schizophrenia spectrum disorder (SSD) is a co-occurrent mental disorder in adults with ASD (Lugo-Marín et al., 2019; Trevisan et al., 2020; Ribolsi et al., 2022). Also, the two conditions share many symptoms. While schizophrenia is characterized by a combination of positive symptoms (delusions and hallucinations), negative symptoms (anhedonia, apathy, social withdrawal) and cognitive symptoms (disorganized thinking, memory difficulties, altered cognitive control) (American Psychiatric Association, 2013; Trevisan et al., 2020; Ribolsi et al., 2022), the negative symptoms, such as impaired social reciprocity, nonverbal communication difficulties, limited gestures, restrictive and repetitive behaviors, social–emotional communication deficits, sensory abnormalities, poor adaptive behavior are also found in ASD (Spek and Wouters, 2010; Wouters and Spek, 2011; Fitzgerald, 2012; Øie et al., 2020). The presence of these symptoms in both disorders could lead to misdiagnosis in some ASD cases. Furthermore, the conceptualization of these two conditions has changed since 2013 with the publication of the DSM-5 (American Psychiatric Association, 2013), whereby both disorders evolved toward a dimensional perspective, replacing the dichotomous approach. This change in perspective comes from the heterogeneous clinical manifestations of symptoms observed in the diagnosis of autism and schizophrenia (Tarasi et al., 2022). Comparing these two disorders, which share significant characteristics, will give us the opportunity to gain a better understanding of the factors that may be related to poor adaptive functioning. Equally important, comparing them will provide us with more precise information about the possible similarities and differences in the roles that EF, IQ, and core ASD symptoms may have on DLS in each disorder.

The nature of our study was exploratory as no other study has used a similar approach to detect specific difficulties related with EFs, IQ, and ASD symptoms over DLS. Our first objective was to examine the relationship between IQ and adaptive behavior, specifically DLS. For this purpose, we assessed IQ to determine the level of adaptive behaviors in our groups with the Vineland-II DLS Domain. We expected that adults with ASD with an average IQ would continue to present important deficits in these type of adaptive behaviors as has been shown in younger ASD individuals (Bertollo and Yerys, 2019). However, there is no evidence that suggests we should find a negative correlation for the SSD group.

Our second objective was to examine the prediction that deficits in EFs could explain the gap between IQ and adaptive behavior in ASD. The novelty of our approach here lies in the use of both objective and subjective measures of EF. To measure EF objectively, we used neuropsychological tasks centered on tapping three EF domains; inhibition, updating, and switching-all of which have been frequently found to be impaired in both ASD and SSD (Spek and Wouters, 2010; Marinopoulou et al., 2016). To measure EF subjectively, we used the Dysexecutive Questionnaire-Spanish (DEX-Sp). This test is a self-report questionnaire that measures people’s perceptions about difficulties they have in everyday life EF, such as situations where they have to remember things, pay attention to certain events while inhibiting others or where they have to stop impulses. With its two subtests, DEX-Sp measures two types of problems: disorganization and apathy subscale (explores difficulties to initiate, engage or maintain a behavior) as well as disinhibition and impulsivity subscale (explores difficulties to inhibit inappropriate responses or behaviors). We compared, ASD, SSD and typically developing adult groups on the DLS and executive functioning. In line with past evidence that shows difficulties in executive functioning, i.e., inhibition, organization, planning, and goal-directed behavior in both ASD and SSD (de Boer et al., 2014; Øie et al., 2020; Shi et al., 2020; Yon-Hernández et al., 2022a), we expect to find a significant correlation between the deficits in EFs and low DLS in both groups. Our study attempts to clarify to what extent the presence of these difficulties in EF affects DLS in both conditions.

The third objective was to assess if the core symptoms of ASD might contribute to lower DLS. To do that, we used different tools that look into the core symptoms of ASD. We used the Autism Diagnostic Observation Schedule-2 (ADOS-2) and the Autism Quotient-Short (AQ-S) to measure social interactions and communication (Criterion A, DSM-V) and the Repetitive Behaviours Questionnaire-2 Adults (RBQ-2A) was used to measure repetitive behavior (Criterion B, DSM-V) in the two clinical groups. While the ADOS is a more objective measure of problems in communication and interaction (because it is scored by the professional), the RBQ-2A is a subjective measure of the presence of repetitive behavior (because it is self-administered questionnaire). The AQ-S, on the other hand, is a subjective measure of symptoms that fall into both A and B criteria. This is the first time, to our knowledge, that both types of instruments have been used in the adult ASD population to assess the effect of how the severity of ASD core symptoms affect DLS. In line with previous research in children with ASD (Kanne et al., 2009) that used multi-informant ratings of psychiatric symptom severity, we expected the presence of core autism symptoms will be important for adaptive functioning, but for the ASD group only.

### Methods

#### Participants

The participants in this study were part of a larger study assessing the role of executive function in ASD and SSD. A clinical questionnaire was obtained from all 61 participants about their medication and previous medical history as well as mental health diagnoses (see Table 1). The inclusion criterion for this study for all groups was to have an IQ of 70 or more. Each group characteristics are described below.

**Table 1:** Sample characteristics.

	Group		
	Mean (SD)		
	TDC (*n* = 25)	ASD (*n* = 24)	SSD (*n* = 12)
Age	28.48 (10.21)	29.38 (11.55)	42.75 (13.16)
Age range	18–63	16–54	21–62
**Psychopharmacological treatment**			
PANSS-P	–	–	10.00 (2.79)
PANSS-N	–	–	12.72 (5.46)
PANSS-GP	–	–	24.27 (3.28)
Antipsychotic	0%	0%	100%
Antidepressant	0%	8.3%	0%
Anxiolytic	0%	4.3%	41.7%
Mood stabilizer	0%	4.2%	16.7%
Methylphenidate	0%	4.2%	0%
**Education level**			
Mandatory school	0%	50%	58.3%
University	100%	50%	41.7%
**Professional status**			
Student	48%	50%	4.5%
Employed	44%	12.5%	0.0%
Unemployed	4%	37.5%	81.9%
Retired	4%	0%	13.6%

TDC, Typical Developmental Controls; ASD, Autism Spectrum Disorder; SSD, Schizophrenia Spectrum Disorder; PANSS, Positive and Negative Syndrome Scale; PANSS-P, Positive and Negative Syndrome Scale-Positive Subscale; PANSS-N, Positive and Negative Syndrome Scale-Negative Subscale; PANSS-GP, Positive and Negative Syndrome Scale-General Psychopathology Subscale.

#### Typical development control group

A total of 25 participants were recruited from the general population and college students. All participants met the IQ criterion, and no participants were excluded for obtaining scores above the cut-off point for the Autism Quotient-Short questionnaire.

#### Autism spectrum disorder group

A total of 24 participants with ASD were included in this group. They received a clinical diagnosis prior to this study. The diagnosis was confirmed with the Autism Diagnostic Observation Schedule (ADOS-2) (Modules 3–4) (Lord et al., 2015) for all participants except for two due to their unavailability. One participant reported having epilepsy and four participants reported taking medication (see Table 1); one participant informed being colorblind and, therefore, could not perform one EF task involving colors.

#### Schizophrenia spectrum disorders group

The schizophrenia spectrum disorders (SSD) group consisted of 15 participants with an SSD diagnosis according to the DSM-5 criteria (American Psychiatric Association, 2013). Psychiatric records indicated no previous history of substance abuse in the 5 years prior to the study (e.g., use of alcohol, cannabis, hallucinogens, or opioids). Inclusion criterion for this group was that no acute psychotic symptoms were present at the time of the study as assessed by the Positive and Negative Syndrome Scale-Spanish version (PANSS) (Kay et al., 1987; Peralta and Cuesta, 1994) (see Table 1). Antipsychotic medication doses were within the guidelines recommended by Spanish drug regulations. Three participants were excluded from the study as they had an IQ < 70.

### Procedures

Informed consent was obtained from all participants and underaged participants signed the assent form. This study was approved by the Bioethical Committee of Universidad de Salamanca. Individual assessments were conducted in two or three sessions by a trained researcher, each with a maximum duration of 60–70 min.

### Materials

#### Wechsler adult intelligence scale-IV

We assessed intelligence with the Wechsler Adult Intelligence Scale-IV (WAIS-IV) (Wechsler, 2012). All mandatory subscales were administered to each participant to obtain a Full-IQ (FIQ) score, Verbal-IQ (VIQ) score and Performance-IQ (PIQ) score (see Table 2).

**Table 2:** Group differences on IQ, EFs and ASD core symptoms.

	Group									
	Mean (SD)									
	TDC (*n* = 25)	ASD (*n* = 24)	SSD (*n* = 12)	*H*	df	*p*	TDC-ASD	TDC-SSD	ASD-SSD	𝜖^2^
FIQ	116.44 (16.72)	107.92 (20.50)	98.83 (17.84)	6.541	2	0.038	–	0.012	–	0.11
VIQ	131.60 (12.85)	121.83 (19.33)	119.83 (21.04)	4.095	2	0.129	–	–	–	0.07
PIQ	106.84 (18.85)	99.71 (22.68)	92.58 (21.18)	4.108	2	0.128	–	–	–	0.07
**ASD Core Symptoms Measures**										
AQ-S	51.16 (6.76)	75.96 (12.39)	63.58 (11.74)	37.062	2	0.001	0.001	0.010	-	0.62
				***U***	***Z***	***p***				
ADOS-2	–	11.32 (2.84)	3.58 (3.92)	16.00	−4.198	0.001				0.53
ADOS-COM	–	4.23 (1.19)	0.83 (1.19)	7.00	−4.604	0.001				0.64
ADOS-SI	–	7.09 (2.39)	2.58 (3.06)	35.00	−3.520	0.001				0.38
ADOS-RRB	–	2.09 (1.34)	0.67 (0.78)	50.00	−3.044	0.002				0.28
				***H***	**df**	***p***	**TDC-ASD**	**TDC-SSD**	**ASD-SSD**	***e***^**2**^
RBQ-2A	1.31 (0.16)	2.03 (0.37)	1.50 (0.45)	31.472	2	0.001	0.001	-	0.004	0.53
**Executive Functions Measures**										
Inhibition	1.24 (0.17)	1.09 (0.26)	1.11 (0.30)	5.010	2	0.082	–	–	–	0.08
Updating	1.23 (0.17)	1.12 (0.28)	0.95 (0.18)	11.683	2	0.003	–	0.002	–	0.20
Shifting	1.33 (0.12)	1.13 (0.32)	0.93 (0.31)	15.873	2	0.001	0.035	0.001	–	0.27
DEX-Sp	12.72 (6.62)	36.25 (11.55)	21.67**(12.55)**	32.820	2	0.001	0.001	–	0.021	0.55
DEX-Sp Disinhibition/Impulsivity	6.40 (3.12)	16.04 (6.40)	11.08 (7.68)	23.469	2	0.001	0.001	–	–	0.56
DEX-Sp Disorganization/Apathy	6.32 (4.43)	20.21 (6.32)	10.58 (6.61)	33.776	2	0.001	0.001	–	0.006	0.39
**Daily Living Skills**										
DLS Domain-VABS	96.64 (10.61)	69.63 (8.20)	79.67 (10.10)	36.665	2	0.001	0.001	0.018	–	0.61

TDC, Typical Developmental Controls; ASD, Autism Spectrum Disorder; SSD: Schizophrenia Spectrum Disorder; FIQ, Full-Scale Intelligence Quotient; VIQ, Verbal Intelligence Quotient; PIQ, Performance Intelligence Quotient; AQ-S, Autism Quotient Short; ADOS, Autism Diagnostic Observation Schedule; COM, Communication; SI, Social Interaction; RRB, Restricted and Repetitive Behavior. H, Kruskal–Wallis *H* test; U, Mann–Whitney *U* test; DF, Degrees of Freedom; Z, *Z*-Score. Significance adjusted with Bonferroni correction *p* = 0.05.

#### Autism diagnostic observation schedule – 2

Both ASD and SSD group were assessed with the Autism diagnostic observation schedule – 2 (ADOS-2) (see Table 2). The ADOS-2 (Lord et al., 2015) is a standardized, semi-structured assessment, which consists of a set of different activities aimed at detecting the presence of unusual social and communicative behavior as well as repetitive and restrictive behavior and sensory issues related to ASD. The ADOS-2 has five different modules used according to age and language level of the person tested (T, 1, 2, 3, and 4). Since participants had a good language level and the fact that we primarily tested adults, modules 3 and 4 were used. The algorithms for ADOS are divided into a Communication score (Comm), Reciprocal Social Interaction score (SI), Stereotyped Behavior and Restricted Interests (RBB) and a Total Score.

#### Autism spectrum quotient – short

The Autism Spectrum Quotient – Short (AQ-S) (Lugo-Marín et al., 2019) is a self-report questionnaire that measures the presence of autistic features in the general population. It has 28 items that assess impairments in social interaction, social communication, imagination, and other cognitive processing in ASD. The cut-off point is >63 for autistic traits. We administered the AQ-S to all the groups.

#### Repetitive behavior questionnaire – 3

The repetitive behavior questionnaire (RBQ-3) is a new version of the Adult Repetitive Behaviour Questionnaire-2 (RBQ-2A) (Barrett et al., 2015; Joyce et al., 2017), which is a self-report questionnaire, suitable for all ages. This tool is intended for measuring restricted and repetitive behavior in the general population. To date, there are no available cut-off scores to indicate clinical abnormality. However, the standard questionnaire scoring enables the comparison of scores between groups. Based on the previous research from the RBQ-2A this scale has a two-factor structure: Factor 1: Repetitive motor behavior (RMB) and Factor 2: Insistence on sameness (IS). The validation study by Barrett et al. (2015) indicated that a neurotypical mean for the total RBQ-2A score was 1.25, for RMB 1.26, and IS 1.29. Meanwhile, for individuals with autism, the mean score was 1.84, on RMB 1.59, and IS 2.04.

#### Neuropsychological tasks

We assessed three core components of EFs: updating, inhibition, and shifting, following the Miyake and Friedman assessment approach (Friedman et al., 2008; Marinopoulou et al., 2016). Updating is the ability to use or maintain information on ongoing behavior and we examined it with Keep-Track, Letter-Memory, and Spatial 2-Back task. Shifting is the ability to switch between one mental activity or action to another and we assessed it with Number-Letter, Color-Shape, and Category-Switch task. Finally, inhibition, which is the ability to suppress unwanted responses and irrelevant information, was examined with Antisaccade, Stop-Signal, and the Stroop task. The tasks were computerized using OpenSesame (Mathôt et al., 2012) and administered in a MacBook Pro 13″. Specific information on the details and design of each task can be found in Yon-Hernández et al. (2022b). We obtained individual scores from these tasks that were later computed as an overall domain score for each EF component.

#### Dysexecutive questionnaire-Spanish

The Dysexecutive Questionnaire-Spanish (DEX-Sp) (Wilson et al., 1996; Llanero Luque et al., 2008) is a 20-item self-report questionnaire that covers different daily living EF problems. It is designed to screen observable, everyday manifestations of executive dysfunctions, such as problems in attention, memory, information processing, behavioral control, emotion regulation and so forth. Scores below 18 points are attributed to individuals without dysexecutive problems, scores ranging from 19 to 28 suggest a moderate dysexecutive problem, and scores above 28 points indicate significant impairments in daily EFs (i.e., Dysexecutive Syndrome-DS). This questionnaire has two subscales: the Disorganization/Apathy subscale and the Disinhibition/Impulsivity subscale. The former subscale comprises items that explore difficulties in initiating or maintaining a behavior as well as in organizing and performing a planned behavior; the latter explores difficulties in inhibiting responses or unwanted behaviors when these are inappropriate for the immediate context.

#### Vineland adaptive behavior scale, second edition

The Vineland adaptive behavior scale, second edition (VABS-II) was designed to measure an individual’s personal, social, and practical competence needed for everyday living across the lifespan (Sparrow et al., 2005). In this study, we used the VABS-II Survey-Interview Form. In the case of adult participants, the VABS-II was administered by the interviewer directly to the adult; in the case of underage participants, the VABS-II was administered to the participants’ parents. The VABS-II has 4 principal domains; however, for the purposes of this study, we only administered the DLS domain, which gathers information on individuals’ ability to take care of themselves, accomplish household chores, or follow community rules, among other practical daily living skills (Sparrow et al., 2005). The DLS domain is constituted by the DLS-Personal subdomain, DLS-Domestic subdomain and DLS-Community subdomain. The standard score for the DSL domain had a mean of 100 and a standard deviation of 15.

### Analysis

Analyses were conducted using SPSS 26.0 (IBM Corp, 2019). The assumptions for conducting a parametric test were not met, therefore, we decided to run non-parametric Kruskal–Wallis *H* test to examine group differences as well as a *post hoc* analysis. Pairwise group comparisons were performed using Dunn’s procedure (Dunn, 1964) with a Bonferroni correction for multiple comparisons. Adjusted *p*-values were reported with significance-level set at <0.05.

We further ran linear regression to understand the effect of IQ scores on the outcomes in the VABS-DLS domain scores in all groups. The assumptions to conduct a linear regression were met for the ASD and Typical development control (TDC) data, yet the assumption for normally distributed data were not met for the SSD group data; nonetheless, we decided to continue the analysis as no other statistical option was available, but we acknowledge this was a limitation.

Individual hierarchical multiple regressions were used to understand the effect our independent variables (ASD core symptoms and EFs) had on our dependent variable (DLS), by considering the potential influence of IQ. Therefore, two models were assessed: in model 1 we studied the effect of IQ in DLS; in model 2 we reduced the confound effect of IQ and separately introduced ASD core symptoms and EFs as individual independent variables. We differentiated the objective and subjective measures. The models were as follows: ASD Core Symptoms Predicting DLS (objective measures): (model 1) IQ = DLS, (model 2) IQ + ADOS + ADOS-COM + ADOS-SI + ADOSRRB = DLS. ASD Core Symptoms Predicting DLS (subjective measures): (model 1) IQ = DLS, (model 2) IQ + AQ-S + RBQ-2A + RBQ-2A-Factor 1 + RBQ-2A-Factor 2 = DLS. EFs Predicting DLS (objective measures): (model 1) IQ = DLS, (model 2) IQ + INHIBITION + UPDATING + SHIFTING = DLS. EFs Predicting DLS (subjective measures): (model 1) IQ = DLS, (model 2) IQ + DEX-Sp + DEX-Sp-Subscale 1 + DEX-Sp-Subscale 2 = DLS.

## Results

Descriptive statistics of the sample characteristics and psychopharmacological use are summarized in Table 1. It is important to acknowledge that 4 individuals from the SSD group obtained scores above the cut-off point for ASD (≥7) according to the ADOS-2 algorithm. Also, five SSD participants scored above the cut-off point on the AQ-S.

### Group sample differences in IQ, ASD core symptoms and EFs

The characterization of our sample is shown in Table 2, as well as the group differences found in our targeted variables.

### Predictive effect of IQ On adaptive skills – DLS

Linear regression in the TDC group showed that the FIQ score and DLS did not have a statistically significant linear relationship [*F*(1,23) = 0.044, *p* > 0.835]. The ASD group, in contrast, reflected a linear regression in which an overall FIQ predicted the DLS outcomes [*F*(1,22) = 8.758, *p* < 0.05] and FIQ accounted for 25% of the explained variability in the adaptive skills outcomes. As for the SSD group, the analyses showed that the FIQ and DLS did not have a linear relationship [*F*(1,10) = 0.651, *p* > 0.438].

As for VIQ, the results indicated that in the TDC group, VIQ and DLS did not have a linear relationship [*F*(1,23) = 0.048, *p* > 0.829]. For the ASD group, it was established that VIQ could predict the outcomes in DLS [*F*(1,22) = 9.166, *p* < 0.05]. The VIQ accounted for 29% of the explained variability in DLS outcomes. As for the SSD group, the linear regression showed that VIQ and DLS did not have a linear relationship [*F*(1,10) = 0.008, *p* > 0.930].

Lastly, linear regression in the TDC group showed that PIQ and DLS did not have a linear relationship [*F*(1,23) = 0.000, *p* > 0.993]. For the ASD group, linear regression established that the score in PIQ statistically predicted the outcomes in DLS [*F*(1,22) = 4.484, *p* < 0.05], whereby, PIQ accounted for 16% of the explained variability in DLS outcomes. As for the SSD group, the linear regression showed that PIQ and DLS did not have a linear relationship [*F*(1,10) = 1.135, *p* > 0.312].

### ASD core symptoms predicting DLS (objective measures)

Hierarchical multiple regressions were conducted for the available data (ASD and SSD groups) regarding ASD core symptoms. However, the analyses showed a similar pattern between the ASD group and SSD group, in which Model 2 (the full model) was not statistically significant and did not predict DLS outcomes {[*R*^2^ = 0.467, *F*(3,15) = 0.565, *p* > 0.647, *R*^2^ adjusted = 0.254] and [*R*^2^ = 0.670, *F*(4,4) = 0.537, *p* > 0.470, *R*^2^ adjusted = 0.091] respectively}.

### ASD core symptoms predicting DLS (subjective measures)

The results from the hierarchical multiple regressions showed that the TDC group and SSD had a similar pattern, in which Model 2 (full model) was not statistically significant and did not predict DLS outcomes for these groups {[*R*^2^ = 0.104, *F*(4,17) = 0.092, *p* > 0.779, *R*^2^ adjusted = −0.265] and [*R*^2^ = 0.581, *F*(4,4) = 1.068, *p* > 0.632, *R*^2^ adjusted = −0.154] respectively}. Meanwhile, in the ASD group, the results demonstrated that Model 2 significantly predicted the DLS outcomes in this group [*R*^2^ = 0.633, *F*(4,16) = 3.092, *p* < 0.05, *R*^2^ adjusted = 0.472].

### EFs predicting DLS (objective measures)

The hierarchical multiple regressions showed a similar pattern between the TDC group and SSD group, in which Model 2 (full model) did not predict DLS outcomes {[*R*^2^ = 0.420, *F*(3,18) = 0.408, *p* > 0.094, *R*^2^ adjusted = 0.227] and [*R*^2^ = 0.510, *F*(3,5) = 0.403, *p* > 0.867, *R*^2^ adjusted = −0.958] respectively}. As for the ASD group, results were statistically significant and indicated that Model 2 predicted DLS outcomes in this group [*R*^2^ = 0.522, *F*(3,17) = 2.053, *p* < 0.05, *R*^2^ adjusted = 0.354].

### EFs predicting DLS (subjective measures)

The results from the hierarchical multiple regressions showed that the TDC group and SSD group had a similar pattern, in which Model 2 (full model), was not significant and did not predict DLS outcomes for these groups {[*R*^2^ = 0.048, *F* (2,19) = 0.362, *p* > 0.962, *R*^2^ adjusted = −0.202] and [*R*^2^ = 0.200, *F*(2,6) = 0.252, *p* > 0.897, *R*^2^ adjusted = −0.467] respectively}. Meanwhile, in the ASD group, the results demonstrated that Model 2 significantly predicted DLS outcomes in this group [*R*^2^ = 0.610, *F*(2,18) = 6,017, *p* < 0.05, *R*^2^ adjusted = 0.502].

## Discussion

The present study aimed to understand how different characteristics may influence adaptive functioning in autism and schizophrenia, two disorders that share a series of symptoms related to impairments in social functioning. Our interest was powered by the idea that the identification of key factors that influence adaptive functioning can help establish therapeutic goals that may lead to the achievement of desired outcomes for adults with ASD and SSD. As predicted, the group with ASD scored significantly lower on daily living skills, revealing significant problems in their daily lives. Participants in the SSD showed low adaptive functioning as well, but not as low as the ASD group. With respect to the ASD group, our findings go in line with vast research that indicates difficulties in adaptive functioning in DLS that occur at all ages (Smith et al., 2012; Franchini et al., 2018; Meyer et al., 2018; Tomaszewski et al., 2020).

Similarly, there is abundant evidence that demonstrates that low IQ is associated with significant difficulties in adaptive functioning (Duncan and Bishop, 2015; McQuaid et al., 2021). However, these are not exclusive to people with ASD and low IQs, as people with ASD who have average IQs also have significant adaptive difficulties. The same pattern was observed in individuals with higher IQs whose adaptive problems were considerable despite their high IQ (Kenworthy et al., 2010; Pugliese et al., 2016; Vogan et al., 2018; Bertollo and Yerys, 2019; Simonoff et al., 2020). Our analysis of the effect of IQ on adaptive functioning pointed in this direction as well. The increased IQ scores were not associated with an improvement in adaptive functioning in our study. More importantly, we identified a significant linear relationship between all IQ values (FIQ, VIQ, and PIQ) and DLS but only for the ASD group, in the sense that a high IQ was associated with low adaptive functioning. This result also indicated that, while in the ASD group there is a clear discordance between high IQ and expected DLS, this is not the case for the SSD and TDC groups. These results suggested that it is not sufficient to consider only IQ as a determining factor in understanding the DLS difficulties of individuals with ASD. This leads us to the conclusion that other factors may play a more important role in adaptive functioning deficits in ASD. The results obtained in this study also support existing findings on the discrepancy between IQ and poor adaptive competence in adulthood, suggesting that the pattern of difficulties in DLS is present throughout development and well into adulthood.

Although the focus of this research was not to study language, we believe it is important to highlight that according to our results, the VIQ alone was responsible for 29% of the variance explained in the scores obtained by the ASD group in adaptive functioning in everyday life skills. Although these results did not fully explain the deficits in adaptive functioning, they accounted for a large percentage of the adaptive deficits seen in these individuals. It is equally important to remember that other studies have pointed to the VIQ as a predictor of successful outcomes in adaptive behavior in different age groups of individuals with ASD and as a variable that may influence the severity of ASD symptoms depending on whether its value is higher or lower (Bal et al., 2019; Hyman et al., 2020). Therefore, in line with other research (Baker et al., 2021), our findings further support the idea that it is not sufficient to have a preserved IQ to ensure that a person with ASD has optimal adaptive functioning.

As argued before, the severity of ASD core symptoms (difficulties in social communication and repetitive behavior) has been proposed as an important factor that could influence lower scores in adaptive functioning in the adult population. Pugliese et al. (2016) suggested that the severity of symptoms could explain a part of the variance in adaptive difficulties for DLS. However, other researchers have found that ASD core features have little predictive value for adaptive difficulties in some ASD groups (Kanne et al., 2009), attributing the variability in their results to differences in the methodology used in the studies or to the type of informant used to report deficits in adaptive behavior. For this reason, in this study we decided to use two different methodologies incorporating an objective instrument (ADOS-2) as well as subjective instruments (RBQ-2A and AQ-S). As expected, the data obtained with the ADOS-2, our objective measure, demonstrated that only the ASD group showed significant greater scores in ASD symptomatology severity. Our data also indicated that there was no association between scores in adaptive functioning and the severity of the core features of ASD, as assessed by the ADOS-2. This result is similar to that obtained by Duncan and Bishop (2015). As for the TDC and SSD groups, no significant association was found neither between difficulties in adaptive functioning, nor the severity of ASD symptoms as measured by the ADOS-2. Regarding the subjective measures of severity of ASD symptoms, we found that the scores of participants with SSD in the AQ-S questionnaire were high, with no significant differences between the scores from this group and the scores of the ASD group. As for the RBQ-2A, we found that there were significant differences between the three groups, participants with ASD having significantly higher scores on this questionnaire. This means that at the time of the study, the ASD group reported a high presence of repetitive and stereotyped behaviors. This contrasted with the scores from the other two groups, as they both obtained lower scores than ASD. More importantly, we found a significant relationship between ASD symptom severity, as measured by the AQ-S and RBQ-2A, and the DLS in the ASD group. Our results support the extensive research on children and adolescents which shows a relationship between ASD symptom severity and adaptive behavior (see Yon-Hernández et al., 2022a). Therefore, the type of measurement use might be important in detecting whether the severity of symptoms influences adaptive functioning. We should also note that the severity of core ASD symptoms seems to play a relevant role in ASD, but not in the SSD group. Future research should continue this line of investigation to improve the use of self-report questionnaires to study possible factors related with EFs in ASD. Additionally, it would also be interesting to study which core symptoms of ASD might play a more influential role in their adaptive functioning because it could help explain the problems experienced by individuals with ASD in everyday life situations (Nakata et al., 2020).

We also analyzed the influence of EF deficits on adaptive functioning in the three groups. We have highlighted that impaired EF in ASD has been associated with adaptive difficulties in everyday tasks (Kenworthy et al., 2008; Pugliese et al., 2016; Baker et al., 2021). The same results have been reported for SSD in terms of impairments in EF and adaptive functioning (Leifker et al., 2009). In this study, the neuropsychological executive functioning tasks used indicated group differences between the TDC and SSD on updating (ability to use or maintain information from ongoing behaviors) and shifting (ability to switch from one mental activity or action to another), whilst group differences in shifting were observed between ASD and TDC. As for the subjective measures, we found that the difficulties reported in the SSD group were moderate. As for the ASD group, they reported that their difficulties in daily executive functioning were significantly greater than those from the SSD group. This pattern is similar to the one reported by Yon-Hernández et al. (2022a) where a more exhaustive analysis of the DEX-Sp (our subjective measure) and its subscales was carried out in a larger sample. When analyzing the DEX-Sp subscales in more detail, we found that more difficulties are observed on the disorganization/apathy scale than disinhibition/impulsivity subscale, possibly due to greater role of the former in the daily living skills in ASD. We concluded in that study that executive functioning impairments pertaining to disorganization/apathy, such as those related to initiation of appropriate behaviors, elaboration of strategies, the ability to organize and plan an action, as well as the ability to initiate goal-directed behaviors, are directly connected to low adaptive functioning in day-to-day activities.

It is noteworthy to mention that, in the current study, for the SSD group, both types of measures (i.e., objective and subjective) failed to detect an effect in which EFs underlie difficulties in adaptive functioning. Our findings on SSD go in line with past studies (Green, 2016); however, these results must be interpreted with caution due to the small size of our sample of participants with SSD. Nevertheless, our findings on ASD are consistent with those found by Pugliese et al. (2016), where adults with ASD and average IQs showed deficits, for example, in inhibition, flexibility skills, and self-control/goal-directed skills. Although these measures were significantly lower in the SSD group compared to the ASD group results, SSD’s scores did not achieve the same predictive value as observed in the ASD group. This suggests that limitations in EF do not have the same relevance in explaining difficulties in adaptive functioning in SSD as they do in ASD.

Given that both SSD and ASD are lifelong disorders, research such as this one is important to shed light on the differences between ASD and SSD and helping determine how to differentially intervene in these populations to improve adaptive behavior. For instance, it seems relevant to incorporate systematic executive functioning information when designing intervention and support systems, to better define therapeutic goals aimed at improving adaptive skills for everyday life tasks. The finding of significant deficits in EF on both subjective and objective measures, and the finding of an association between these measures and adaptive functioning supports this suggestion. As noted by Baker et al. (2021), interventions during adolescence can lay the foundation for adulthood and future independent life skills in ASD. Interventions during this period of transition into adulthood should, therefore, include recurrent and frequent executive functioning training in everyday settings to improve adaptive skills and other adaptive behavior, as suggested by this study.

### Limitations

Our SSD sample was small, and thus our results may not be fully generalizable. Additionally, we believe that future research should assess other influential factors such as the role of medication, age, duration of the illness, or the number of psychotic episodes until reaching psychopharmacological stability.

## Data availability statement

The original contributions presented in the study are included in the article, further inquiries can be directed to the corresponding author.

## Ethics statement

The studies involving human participants were reviewed and approved by Bioethical Committee of Universidad de Salamanca. Written informed consent to participate in this study was provided by the participants’ legal guardian/next of kin.

## Author contributions

JY-H, DW, and RC-B participated in the conceptualization, methodology, study design, data collection, and statistical analyses. CF-A participated in the data collection and elaboration of the manuscript. SP-D participated in the elaboration of the manuscript. MF-M and LG-G participated in the recruitment and data collection from the SSD sample. RC-B and MF-M provided resources and funding acquisition. All the authors have reviewed and approved the final manuscript.

## Funding

This study was part of the Ph.D. project of JY-H and funding was granted by the University of Salamanca and Banco Santander. We declare that the funding body did not participate in the design of the study, collection of data, analysis, interpretation or writing the manuscript.

## Conflict of interest

The authors declare that the research was conducted in the absence of any commercial or financial relationships that could be construed as a potential conflict of interest.

## Publisher’s note

All claims expressed in this article are solely those of the authors and do not necessarily represent those of their affiliated organizations, or those of the publisher, the editors and the reviewers. Any product that may be evaluated in this article, or claim that may be made by its manufacturer, is not guaranteed or endorsed by the publisher.
